# Serum from Jiao-Tai-Wan treated rats increases glucose consumption by 3T3-L1 adipocytes through AMPK pathway signaling

**DOI:** 10.1042/BSR20181286

**Published:** 2019-04-05

**Authors:** Lin Yuan, Peng Tang, Hui-Jiao Li, Na Hu, Xiao-Yu Zhong, Min Lin, Yin-Qiang Sun, Min Lu, Xiong Lu

**Affiliations:** Experiment Center for Science and Technology, Shanghai University of Traditional Chinese Medicine, Shanghai 201203, China

**Keywords:** AMPK, Glucose uptake, GLUT4, Insulin resistance, Jiao-Tai-Wan Decoction

## Abstract

Type 2 diabetes (T2DM) is characterized by hyperglycemia resulting from insulin resistance. Jiao-Tai-Wan (JTW), a traditional Chinese medicine consisting of a 10:1 formulation of *Rhizoma Coptidis* (RC) and *Cortex Cinnamomi* (cinnamon) was shown to have hypoglycemic efficacy in a type 2 diabetic mouse model. Here we investigated whether glucose consumption by insulin-resistant adipocytes could be modulated by serum from JTW-treated rats, and if so, through what mechanism. JTW-medicated serum was prepared from rats following oral administration of JTW decoction twice a day for 4 days. Fully differentiated 3T3-L1 adipocytes – rendered insulin resistance by dexamethasone treatment – were cultured in medium containing JTW-medicated rat serum. JTW-medicated serum treatment increased glucose uptake, up-regulated levels of phosphorylated adenosine 5′-monophoshate-activated protein kinase (p-AMPK), and stimulated expression and translocation of glucose transporter 4 (GLUT4). JTW-medicated serum induced significantly greater up-regulation of p-AMPK and GLUT4 than either RC or cinnamon-medicated serum. JTW-medicated serum induced effects on 3T3-L1 adipocytes could be partially inhibited by treatment with the AMPK inhibitor compound C. In conclusion, JTW-medicated serum increased glucose consumption by IR adipocytes partially through the activation of the AMPK pathway, and JTW was more effective on glucose consumption than either RC or cinnamon alone.

## Introduction

Diabetes is a chronic metabolic disease characterized by high blood glucose, mainly the result of a lack of insulin production and/or insulin resistance (IR). Long-term hyperglycemia can damage multiple organs including the heart, eyes, and kidneys. Approximately 90–95% of diabetic patients suffer from type 2 diabetes (T2DM), the pathogenesis of which is due to reduced sensitivity of peripheral tissues to insulin. Incidence and prevalence of T2DM have increased markedly over the past few decades and there is now an urgent need to develop effective therapeutic strategies and drugs that exhibit fewer side effects.

Jiao-Tai-Wan (JTW), a famous traditional Chinese medicine (TCM), is a 10:1 formulation of *Rhizoma Coptidis* (RC) and *Cortex Cinnamomi* (cinnamon). Our previous studies showed that JTW treatment lowered blood glucose and lipid levels and improved insulin sensitivity in the db/db type 2 diabetic mouse model. Furthermore, the effects of JTW were more significant than those of component drugs RC or cinnamon used alone [1]. RC and its principal active constituent berberine are well-known for their effects in treating diabetes and related complications [2]. TCM theory suggests that there are synergistic effects of the constituent herbs of a therapeutic formulation. We were therefore interested in further examining the effects of the JTW formulation of RC and cinnamon in treating diabetes and gaining insights into therapeutic mechanisms.

Adenosine 5′-monophoshate-activated protein kinase (AMPK), a cellular energy sensor, plays an important role in regulating energy balance at both the cellular and whole organism levels. In this role, phosphorylation of the AMPK catalytic α-subunit at Thr-172 is absolutely required for activity [3]. Activated AMPK switches on catabolic activities that increase adenosine triphosphate (ATP), including inhibiting gluconeogenesis in the liver and increasing glucose uptake by muscles and adipocytes, which lowers plasma glucose [4]. The glucose transporter 4 (GLUT4), highly expressed in adipose tissue and skeletal muscle, is responsible for glucose uptake from the circulation. In response to insulin and other stimuli, GLUT4 protein undergoes translocation from the cytoplasm to the plasma membrane to enable glucose transport into the cell. GLUT4 expression and translocation are clearly affected in adipocytes and muscle cells of insulin-resistant (IR) and diabetic patients [5].

In this study we treated IR 3T3-L1 adipocyte cell cultures with serum collected from rats treated with JTW, RC, cinnamon or metformin and measured glucose consumption, AMPK phosphorylation, and GLUT4 expression and translocation. JTW-medicated serum was significantly more effective than either RC or cinnamon-medicated sera in our assays. In addition, using AMPK agonists and inhibitors on cultured 3T3-L1 adipocyte cells we implicated the AMPK pathway in JTW-medicated serum-induced glucose consumption and related effects.

## Materials and methods

### Cells and reagents

Mouse embryonic fibroblast 3T3-L1 pre-adipocytes were obtained from the Cell Bank of the Chinese Academy of Sciences (Shanghai, China). High-glucose DMEM, fetal bovine serum (FBS) and BSA were purchased from Invitrogen. Insulin, 3-Isobutyl-1-methylxanthine (IBMX), Oil Red O dye, AICAR (5-Aminoimidazole-4-carboxamide1-β-D-ribofuranoside), compound C were obtained from Sigma Chemical Co. Antibodies against AMPK α (phosphoT172), AMPK, GLUT4, and GAPDH were purchased from Abcam. Dexamethasone (DEX) and Alexa Fluor546-conjugated secondary antibody were purchased from Life Technologies. RC and cinnamon were purchased from Yanghetang Decoction Pieces Limited Company (Shanghai, China) and extracted by the Analyses and Testing Laboratory of Shanghai University of Chinese Medicine. Metformin was purchased from Sino-US Shanghai Squibb Pharmaceutical Co., Ltd.

### Preparation of JTW, RC, and cinnamon formulations

The RC:cinnamon ratio in the JTW decoction was 10:1. JTW, RC, and cinnamon were prepared as described previously [1]. Briefly, RC was boiled to obtain a water extraction, which was evaporated by heating to a concentration of 0.76 g/ml. Cinnamon was prepared by simultaneous distillation and extraction (SED) to obtain a volatile oil and water extract, which was evaporated to a concentration of 0.076 g/ml at low heat. For JTW decoction, remaining water extract and gruffs of cinnamon after oil extraction were boiled with RC. The extraction solution was filtered and evaporated by heating to a concentration of 0.84 g/ml. Metformin tablets were ground into powder and dissolved in distilled water to a concentration of 0.03 g/ml (metformin’s concentration).

### Preparation of medicated sera

Fifty male Sprague–Dawley rats weighing 250–300 g were purchased from SLAC LTD, Shanghai, China. Animals were housed in plastic cages at 22 ± 1°C with a 12 h light–dark cycle. Food and water were available *ad libitum*. The experiment was approved by the Animal Ethics Committee of Shanghai University of TCM and performed in accordance with the recommendations in the Guide for the Care and Use of Animals. Animals were randomly divided into JTW (*n*=10), RC (*n*=10), cinnamon (*n*=10), metformin (*n*=10), and negative control (*n*=10) groups. JTW, RC, cinnamon, and metformin group animals were administered intragastrically JTW, RC, cinnamon or metformin, respectively, twice a day for 4 days. Negative control animals were administered intragastrically saline on the same schedule. 1 h after the final treatment, rats were intraperitoneally anesthetized using pentobarbital sodium. Blood was collected from the abdominal aorta and centrifuged. Serum samples from all individual animals of each group were pooled, filtered through a 0.22 μm filter membrane, heat-inactivated at 56°C for 30 min, and stored at −80°C until use.

### Cell culture and induced differentiation

3T3-L1 preadipocytes were grown in high-glucose DMEM supplemented with 10% FBS, 50 U/ml penicillin, and 50 μg/ml streptomycin at 37 ˚C in 5% CO_2_ atmosphere with relative humidity of 85–95%. When cells reached 80% confluence, they were induced to differentiate by addition of standard differentiation ‘cocktail’ (DMI; 10 μg/ml insulin, 1 μM DEX, and 0.5 mM IBMX) to the culture medium for 48 h. The medium was then replaced with high-glucose DMEM containing 10% FBS, 10 μg/ml insulin, 50 U/ml penicillin, and 50 μg/ml streptomycin for another 48 h. Subsequently, the cells were maintained in high-glucose DMEM containing 10% FBS, 50 U/ml penicillin, and 50 μg/ml streptomycin until the cells were fully differentiated. Lipid accumulation in the differentiated 3T3-L1 cells was evaluated using OilRedO dye and >90% of the cells exhibited an adipocyte phenotype.

### Induced insulin-resistance and treatment with medicated sera

To induce insulin resistance in adipocytes, 1 μM DEX was added to the fully differentiated adipocyte culture medium for 48 h [6]. A glucose consumption assay was performed to confirm insulin resistance. IR adipocytes were cultured for 48 h in medium containing 10% medicated serum from rats treated with JTW, RC, cinnamon, metformin or saline. To test the effects of AMPK agonist AICAR and inhibitor compound C, 2 mmol/l AICAR or 50 μM compound C were added together with JTW-medicated serum for 48 h.

### Cell viability assay

Cells were seeded into 96-well microplates at 1 × 10^4^ cells/well in 200 μl medium and rendered insulin resistance as described above. Following serum starvation for 3 h, JTW or control saline sera (at concentrations of 5, 10, 20, and 40%) were added to the culture medium for 48 h. The culture medium was then removed and 100 μl of tetrazolium dye MTT (5 mg/ml in PBS) was added to the cells and incubated for 4 h. Subsequently, the MTT solution was removed and 100 μl of DMSO was added to dissolve the formazan crystals. The absorbance value was read at 490 nm by using a microplate reader.

### Glucose consumption assay

After serum starvation for 3 h, IR adipocytes were cultured with medicated serum and/or AMPK agonist/inhibitor for 48 h. Following that the cells were treated with 100 nM insulin for additional 1 h, the culture medium was then collected and the glucose concentration was determined by using the glucose oxidase-peroxidase (GOD-POD) method [7]. 100 μl of culture medium from each well of the culture dish was combined with 900 μl of GOD-POD enzyme solution and incubated at 37°C for 5 min. The optical density at 505 nm was determined by using a spectrophotometer. Glucose consumption by cells cultured with medicated serum and/or agonist/inhibitor was determined by comparing the glucose concentration to the concentration in culture medium of cells cultured in control medium.

### Immunofluorescence assay

3T3-L1 adipocytes were cultured on glass coverslips with medicated serum and/or AMPK agonist/inhibitor for 48 h. Cells were then treated with 100 nM insulin for 1 h, washed with PBS, fixed in 4% paraformaldehyde for 30 min followed by 0.5% Triton X-100 in PBS for 10 min at room temperature. Cells were blocked with 5% BSA solution for 30 min and incubated overnight at 4°C with anti-GLUT4 antibody (1:1000) in BSA. Alexa Fluor 546-conjugated secondary antibody (1:500) was added for 1 h at room temperature. Nuclei were stained with 4′,6-diamidino-2-phenylindole (DAPI) for 5 min. Negative controls lacked primary antibody. Coverslips were mounted on glass slides with antifade mounting medium. Immunolabeled cells were visualized using a Leica SP8 confocal laser scanning microscope.

### Western blotting analysis

Total cellular proteins were obtained by lysing and sonicating cells in ice-cold RIPA buffer containing protease inhibitors, phenylmethanesulfonyl fluoride, and phosphatase inhibitor. 20 μg of total protein was separated by 10% SDS–PAGE and transferred to a polyvinylidene difluoride (PVDF) membrane. After washing, the membranes were blocked using 5% BSA in Tris buffered saline with 0.1% Tween-20 (TBST) for 1 h at room temperature. Membranes were incubated with primary antibodies to total AMPK, phospho-AMPK (Thr172), GLUT4 (1:1000), or GAPDH (1:10000) overnight at 4°C. After washing with TBST, the membranes were further incubated with horseradish peroxidase-conjugated secondary antibodies (1:10,000) in TBST for 2 h. Labeled protein bands were visualized using an enhanced chemiluminescent substrate with the FluorChem E system. Band intensity was quantitatively determined using Image-J software.

### Statistical analysis

The data were expressed as mean ± standard deviation ($\bar{x}\pm \text{SD}$). Data were analyzed using SPSS version 18.0. *T*test and one way ANOVA were used for general data analysis. The LSD method was applied for comparisons between groups. *P*<0.05 was considered statistically significant.

## Results

### JTW-medicated serum increased glucose consumption by IR 3T3-L1 adipocytes

**Figure 1:**
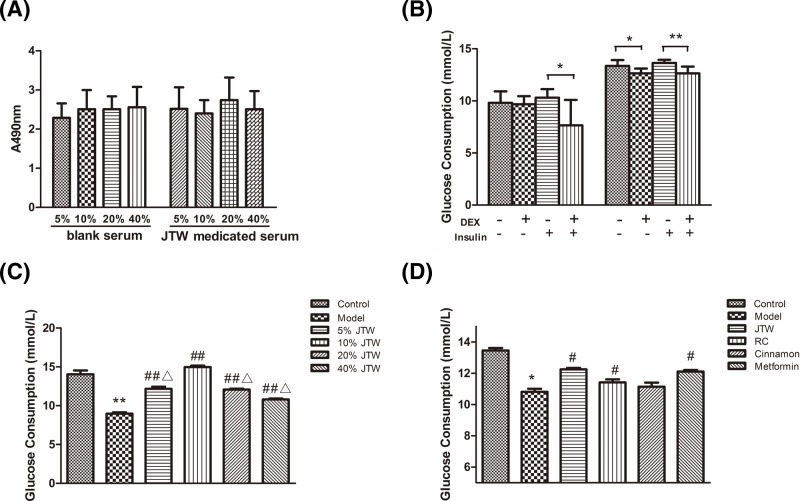
JTW-medicated serum increased glucose consumption in 3T3-L1 adipocytes (**A**) JTW-medicated serum effects on 3T3-L1 adipocyte viability determined by MTT assay. (**B**) Glucose consumption by 3T3-L1 adipocytes treated with 1 μM DEX and insulin for 48 and 96 h. (**C**) Glucose consumption by IR 3T3-L1 adipocytes treated with 5, 10, 20 and 40% JTW-medicated or saline control sera. (**D**) Glucose consumption by IR 3T3-L1 adipocytes treated with 10% JTW, RC, cinnamon or metformin-medicated sera. Data are mean ± SD, *n*=8. ^*^P<0.05, ^**^P<0.01 compared with control serum; ^#^*P*<0.05, ^##^*P*<0.01 compared with model group cells; ^Δ^*P*<0.05 compared with 10% JTW group cells.

We first tested whether exposure to JTW-medicated serum affected cell viability. We performed MTT assays on 3T3-L1 adipocytes cultured in JTW-medicated sera at concentrations ranging from 5 to 40% or negative control serum for 48 h. No significant effect on cell viability was observed as a result of exposure to JTW-medicated serum (Figure 1A).

Fully differentiated adipocytes were treated with 1 μM DEX for 48 and 96 h to induce insulin resistance, leading to significantly reduced glucose consumption after insulin stimulation (*P*<0.01) compared with control cells (Figure 1B). IR adipocytes treated with DEX for 48 h were cultured in JTW-medicated or saline control sera at concentrations of 5, 10, 20, and 40% for an additional 48-h period. JTW-medicated serum treatment increased glucose consumption, with 10% serum treatment leading to a 60% increase (*P*<0.01) relative to control serum treatment (Figure 1C). 10% serum concentrations were therefore used in subsequent experiments.

Next, we compared the effects on glucose consumption of JTW-medicated serum treatment versus RC or cinnamon-medicated sera alone. Metformin-medicated serum was used as a positive control. As shown in Figure 1D, treatment with 10% JTW, RC, and metformin-medicated sera each significantly increased glucose consumption (*P*<0.05) while the effect of cinnamon-medicated serum was not significant relative to negative control saline serum.

### JTW-medicated serum activated AMPK and increased expression of GLUT4 in IR 3T3-L1 adipocytes

**Figure 2:**
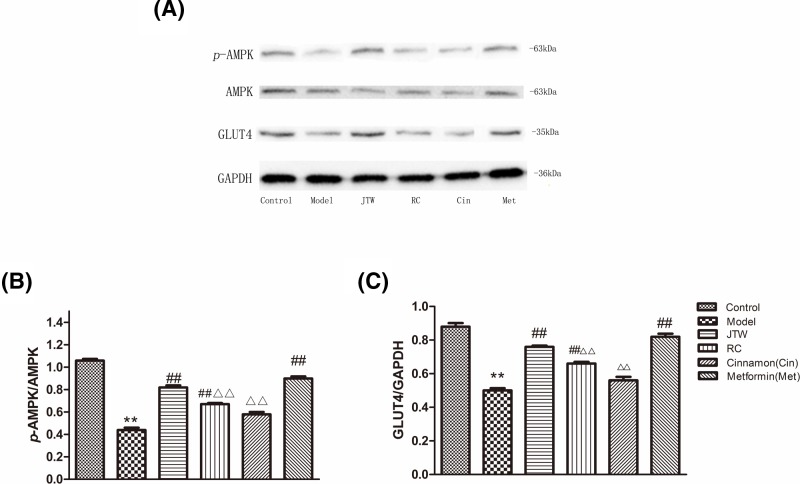
JTW-medicated serum activated AMPK and increased expression of GLUT4 in IR 3T3-L1 adipocytes (**A**) Western blot was performed to detect AMPK and GLUT4 expression levels in JTW-medicated serum-treated IR 3T3-L1 adipocytes. (**B**) Quantification for the p-AMPK level shown in A. (**C**) Quantification for the GLUT4 level shown in A. Data are mean ± SD. ^**^P<0.01 compared with control group cells. ^##^*P*<0.01 compared with model group cells. ^ΔΔ^*P*<0.01 compared with JTW-medicated serum group cells.

AMPK is a key regulator of energy balance. To investigate the mechanism by which JTW-medicated serum increased glucose consumption, we assessed its effects on activation and expression of AMPK. The data showed that phosphorylated AMPK (p-AMPK) levels were decreased in IR adipocytes exposed to untreated control serum (model group) though total AMPK expression was not significantly altered (*P*<0.01). Addition of JTW-medicated serum for 48 h led to increased expression of p-AMPK in IR 3T3-L1 adipocytes compared with the model group (*P*<0.01) (Figure 2A,B). Furthermore, JTW-medicated serum treatment increased the ratio between p-AMPK and AMPK more than either RC or cinnamon sera, though RC-medicated serum also significantly increased activation of AMPK (*P*<0.01) (Figure 2A,B).

GLUT4 plays an important role in glucose transport and expression of GLUT4 was significantly reduced in IR 3T3-L1 adipocytes (*P*<0.01) (Figure 2A,C). We tested the effect of JTW-medicated serum treatment on GLUT4 expression. The data showed that JTW, RC, and metformin-medicated sera each increased GLUT4 expression (*P*<0.01). A significantly greater increase was induced by JTW (*P*<0.01) compared with either RC or cinnamon-medicated sera alone (Figure 2A,C). These results indicated that the JTW formulation of RC and cinnamon was more potent in inducing AMPK activation and GLUT4 expression than either component compound alone.

**Figure 3:**
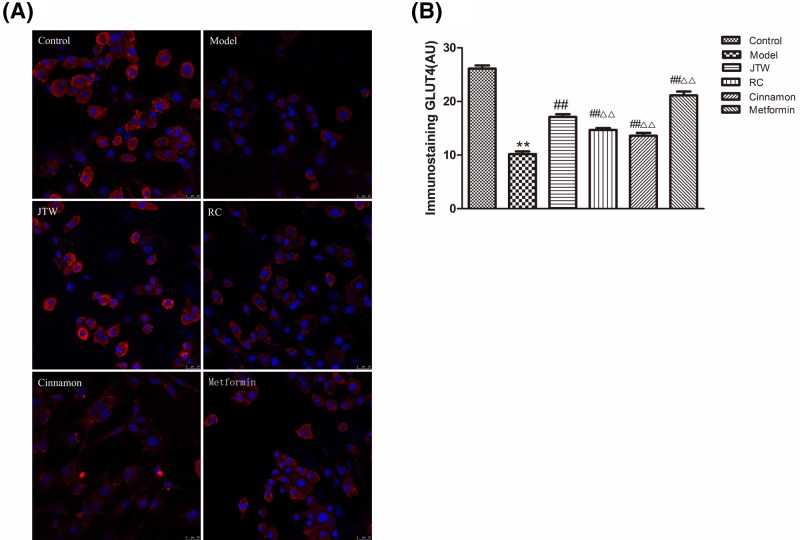
JTW-medicated serum effects on GLUT4 expression and localization in IR 3T3-L1 adipocytes (**A**) Immunostaining was performed to examine the expression and localization of GLUT4 in JTW-medicated serum-treated IR 3T3-L1 adipocytes. Scale bar: 25 µm. (**B**) Quantification for the immunostaining result in A. Data are mean ± SD. ^**^P<0.01 compared with control group cells; ^##^*P*<0.01 compared with model group cells; ^ΔΔ^*P*<0.01 compared with JTW-medicated serum treated group cells.

The translocation of GLUT4 from the cytoplasm to the plasma membrane is critical for glucose transport into the cell. We used immunofluorescence localization to study GLUT4 in IR adipocytes with and without treatment with JTW, RC, cinnamon, and metformin-medicated sera (Figure 3A,B). First, GLUT4 localization was determined in 3T3-L1 adipocytes incubated with insulin for 1 h. IR adipocytes (treated with DEX, model group) exhibited decreased GLUT4 expression (*P*<0.01) compared with controls (cells not rendered IR by DEX treatment). JTW, RC, cinnamon, and metformin-medicated sera each markedly up-regulated GLUT4 expression, with JTW-medicated serum exhibiting more obvious effects than either RC or cinnamon sera (*P*<0.01). Furthermore, GLUT4 showed increased plasma membrane localization after JTW-medicated serum treatment (Figure 3A). These observations confirm JTW-medicated serum promoting GLUT4 expression and also insulin-induced translocation of GLUT4 in IR adipocytes.

### JTW-medicated serum induced glucose consumption by IR 3T3-L1 adipocytes mediated in part by the AMPK pathway

**Figure 4:**
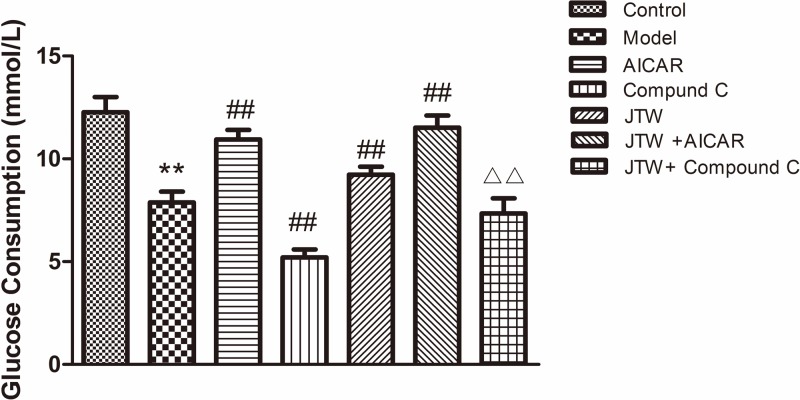
Effects of AICAR and compound C on glucose consumption by IR 3T3-L1 adipocytes treated with JTW-medicated serum Data are mean ± SD. ^**^P<0.01 compared with control group cells; ^##^*P*<0.01 compared with model group cells;^ ΔΔ^*P*<0.01 compared with compound C group cells.

We used an AMPK agonist and an inhibitor to determine whether the effect of JTW-medicated serum on glucose uptake was mediated by the AMPK pathway (Figure 4). The AMPK agonist AICAR increased glucose consumption by IR adipocytes (*P*<0.01). AICAR plus JTW-medicated serum exhibited slightly increased glucose consumption by IR 3T3-L1 adipocytes compared with AICAR alone. The AMPK inhibitor compound C caused decreased glucose consumption by IR adipocytes (*P*<0.01) and partially attenuated the increased glucose consumption induced by JTW-medicated serum (*P*<0.01) (Figure 4). These results indicate that the AMPK pathway is involved in mediating enhanced glucose consumption induced by JTW-medicated serum, but this effect is not entirely dependent on AMPK activity.

### JTW-medicated serum induced GLUT4 expression in IR 3T3-L1 adipocytes partly mediated by the AMPK pathway

**Figure 5:**
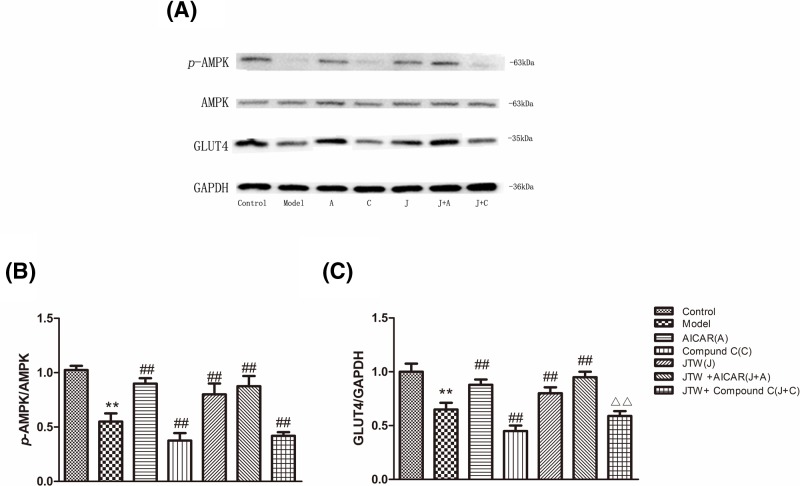
Effects of AICAR and compound C on up-regulation of *p*-AMPK and GLUT4 expression in IR 3T3-L1 adipocytes induced by JTW-medicated serum (**A**) Western blot was performed to examine the effects of AICAR and compound C on p-AMPK and GLUT4 in JTW-medicated serum treated IR 3T3-L1 adipocytes. (**B**) Quantification for the p-AMPK level shown in A. (**C**) Quantification for the GLUT4 level shown in A. Data are mean ± SD. ^**^P<0.01 compared with control group cells; ^##^*P*<0.01 compared with model group cells; ^ΔΔ^*P*<0.01 compared with compound C group cells.

We further tested the effects of AMPK agonist AICAR and inhibitor compound C on the expression of GLUT4 in IR 3T3-L1 adipocytes treated with JTW-medicated serum (Figure 5A). Addition of AICAR to the culture medium with or without JTW-medicated serum led to increased expression of p-AMPK and addition of inhibitor compound C decreased p-AMPK levels. Effects on GLUT4 expression mirrored AMPK activation. Addition of compound C did not entirely attenuate JTW-medicated serum-induced up-regulation of GLUT4 expression (*P*<0.01) (Figure 5A–C).

**Figure 6:**
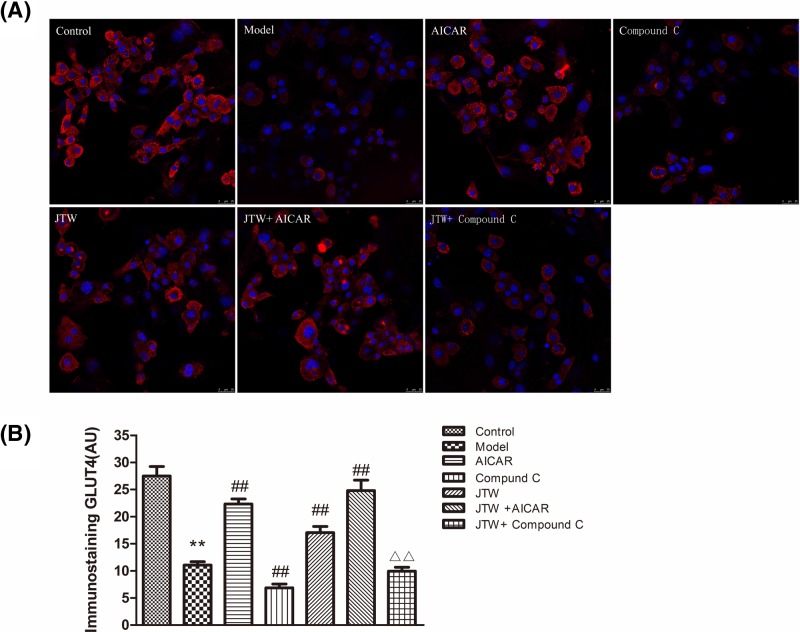
Effects of AICAR and compound C on GLUT4 expression and localization in IR 3T3-L1 adipocytes treated with JTW-medicated serum (**A**) Immunostaining was performed to examine the effects of AICAR and compound C on the expression level and localization of GLUT4 in JTW-medicated serum-treated IR 3T3-L1 adipocytes. Scale bar: 25 µm. (**B**) Quantification for the immunostaining result in A. Data are mean ± SD. ^**^P<0.01 compared with control group cells; ^##^*P*<0.05 compared with model group cells; ^ΔΔ^*P*<0.01 compared with compound C group cells.

JTW-medicated serum increased the expression and translocation of GLUT4 in IR 3T3-L1 adipocytes and led to increased glucose consumption. We next studied expression and localization of GLUT4 protein in IR 3T3-L1 adipocytes co-cultured with JTW-medicated serum plus AMPK agonist or inhibitor (Figure 6A,B). The agonist AICAR alone stimulated GLUT4 expression and translocation, but adding JTW-medicated serum did not further significantly increase GLUT4 expression. The inhibitor compound C partially attenuated the effect of JTW-medicated serum, similar to results obtained from Western blotting experiments. We also observed that translocation of GLUT4 to the plasma membrane after insulin stimulation in JTW-medicated serum treated cells was decreased by the addition of compound C (Figure 6A).

## Discussion

Adipocytes play important roles in the regulation of carbohydrate and lipid metabolism. Numerous studies have shown that abnormal functioning of adipose tissue contributes to the development of insulin resistance and T2DM in rodent models and in humans [8,9]. Our previous studies indicated that the TCM JTW can improve glucose metabolism and insulin sensitivity in the db/db type 2 diabetic mouse model and that the mechanism may be related to activation of the AMPK pathway in liver, muscle, and adipocytes [1]. Here we investigated the effect of JTW-medicated serum on glucose consumption by 3T3-L1 adipocytes *in vitro*, a well-characterized cell culture model for the study of glucose and lipid metabolism. Our results showed that JTW-medicated serum significantly enhanced glucose consumption in differentiated IR 3T3-L1 adipocytes, and activation of the AMPK pathway might contribute to such effect. Compared with RC or cinnamon-medicated sera, JTW-medicated serum is significantly more effective on glucose consumption and AMPK activation.

AMPK is a ubiquitous serine/threonine protein kinase that is involved in various biological processes including glucose homeostasis, lipid metabolism, and inflammation. It is activated in response to low cellular energy status (AMP:ATP ratio). In this role, it contributes to increased glucose uptake, fatty acid oxidation, mitochondrial biogenesis, and autophagy while suppressing the synthesis of fatty acids, cholesterol, and protein [10]. Some hormones including insulin and adiponectin as well as drugs such as metformin and troglitazonecan influence AMPK activity regardless of the cellular AMP:ATP ratio [11]. It has been reported that dysregulation of AMPK is a central contributor to IR-mediated diabetes [4] and AMPK is therefore an important target of treatment strategies for insulin resistance and T2DM. Our current studies demonstrate that JTW-medicated serum can increase the ratio of phosphorylated AMPK to total AMPK in IR adipocytes, which is consistent with our previous experimental results showing that JTW can activate AMPK in white adipose tissue of db/db mice.

GLUT4 is a major transporter for glucose uptake and metabolism in insulin-responsive tissues. Under the stimulation of insulin, GLUT4 proteins are translocated from intracellular stores to the cell membrane to transport glucose for the synthesis of glycogen and fatty acids in skeletal muscle and adipocytes [12]. Both total intracellular GLUT4 and insulin-stimulated GLUT4 translocation are critical factors in the maintenance of glucose homeostasis. Selective overexpression of GLUT4 in fat tissue enhances whole body insulin sensitivity and glucose tolerance in diabetic mice [13]. In this study, we showed that JTW-medicated serum significantly increased GLUT4 expression in IR adipocytes in the absence or presence of 100 nM insulin. Immunofluorescence analysis showed that GLUT4 was predominantly localized to the cell surface in insulin-stimulated cells.

To determine whether JTW-medicated serum effects on glucose consumption by IR adipocytes were mediated by the AMPK pathway, we tested effects of AMPK agonist AICAR and inhibitor compound C on adipocytes treated with JTW-medicated serum. AICAR can be taken up by cells and phosphorylated to yield 5′-aminoimidazole-4-carboxamide ribonucleotide (ZMP), an analog of AMP that activates AMPK without disturbing cellular adenine nucleotide ratios [14]. Compound C is a cell-permeable reversible ATP-competitive inhibitor of AMPK that lowers AMPK phosphorylation [15]. We found that AICAR stimulated glucose utilization and activation of AMPK in IR adipocytes and co-incubation with AICAR plus JTW-medicated serum further increased glucose consumption and GLUT4 expression. Consistent with these observations, AMPK inhibitor compound C partially attenuated enhanced glucose consumption and GLUT4 expression induced by JTW-medicated serum. These results indicate that JTW-medicated serum effects are mediated in part by the AMPK pathway, but additional pathways may also contribute to induction of enhanced glucose consumption by JTW-medicated serum.

GLUT4 expression and function are regulated by multiple signaling pathways, including AMPK pathway and insulin pathway [16]. Myocyte enhancer factor 2 (MEF2) is required for transcription of the *glut4* gene and its activity is regulated by histone deacetylase 5 (HDAC5). AMPK can phosphorylate HDAC5 and promote its dissociation from MEF2, thereby stimulating GLUT4 protein expression [17]. The intracellular insulin signaling pathway is mainly responsible for GLUT4 translocation, an acute response to insulin stimulation. The binding of insulin to its receptor leads to activation of the phosphoinositide 3-kinase (PI3K)/protein kinase B (Akt) pathway, which in turn promotes phosphorylation of the Rab GTPase-activating protein AS160 and leads to translocation of GLUT4 from intracellular storage vesicles to the plasma membrane [18]. During exercise and hypoxia, the AMPK pathway is also thought to play a role in promoting GLUT4 translocation through phosphorylation of the 160 kDa Akt substrate [19,20]. Mirroring effects on glucose consumption, AMPK inhibitor partially reversed the effect of JTW-medicated serum on GLUT4 expression, though serum addition significantly elevated GLUT4 expression (and glucose consumption) relative to compound C without JTW-medicated serum. Thus, the effect of JTW-medicated serum on GLUT4 is partially dependent on the AMPK pathway.

As an important complementary and alternative therapy, TCM is widely used in clinical practice of Chinese medicine. TCM posits synergistic relationships among constituent ingredients formulated in specific proportions; therefore, prescriptions with multiple ingredients have advantages of more efficacies and less toxicity. However, the mechanisms underlying the compatibility and synergy of constituent ingredients still remain unclear. Unlike western medicine, herbal medicine contains various active components that work synergistically and often target to multiple pathways with more pharmacological potency and/or fewer side effects [21] Different TCM herbs in the prescription may have different absorption, distribution, and metabolic characteristics, thus affecting the bioavailability of the whole prescription [22]. RC has been employed in TCM for thousands of years to treat ‘XiaoKe Disease’, a general pathology characterized by polyphagia, polydipsia, polyuria and weight loss, symptoms similar to those of T2DM. Recent studies indicated that RC and its major active component berberine possess multispectrum therapeutic activities, including anti-hyperglycemia, anti-hyperlipidemia, anti-hypertension, anti-inflammatory, and antioxidant effects. Cinnamon has also been shown to affect hypoglycemia [4]. Our observations indicate that compared with RC or cinnamon alone, appropriately formulated JTW indeed exhibited significantly greater effects on glucose consumption, activation of AMPK, and expression of GLUT4 in IR adipocytes *in vitro*. These results justify further studies of JTW decoction as an approach to treat diabetes mellitus and the mechanisms underlying the interaction between RC and cinnamon.

## Abbreviations

AICAR: 5-Aminoimidazole-4-carboxamide 1-β-D-ribofuranoside
AMPK: adenosine 5′-monophoshate-activated protein kinase
cinnamon: *Cortex Cinnamomi*
DEX: dexamethasone
GLUT4: glucose transporter 4
GOD-POD: glucose oxidase-peroxidase
IBMX: 3-isobutyl-1-methylxanthine
IR: insulin-resistant
JTW: Jiao-Tai-Wan
MTT: 3-(4,5-dimethylthiazol-2-yl)-2,5-diphenyltetrazolium bromide
p-AMPK: phosphorylated AMPK
RC: *Rhizoma Coptidis*
T2DM: type 2 diabetes
